# Developing a Mobile App for Young Adults with Nonsuicidal Self-Injury: A Prototype Feedback Study

**DOI:** 10.3390/ijerph192316163

**Published:** 2022-12-02

**Authors:** Kaylee Payne Kruzan, Madhu Reddy, Jason J. Washburn, David C. Mohr

**Affiliations:** 1 Department of Preventive Medicine, Feinberg School of Medicine, Northwestern University, Chicago, IL 60611, USA; 2 Department of Informatics, Donald Bren School of Information & Computer Sciences, University of California Irvine, Irvine, CA 92697, USA; 3 Department of Psychiatry and Behavioral Sciences, Feinberg School of Medicine, Northwestern University, Chicago, IL 60611, USA

**Keywords:** nonsuicidal self-injury, self-harm, mobile app, digital mental health, intervention, young people

## Abstract

Nonsuicidal self-injury (NSSI) affects approximately 13% of young adults. Though evidence-based treatments for NSSI exist, most young adults do not receive treatment. Digital interventions can provide access to evidence-based treatments for NSSI at scale. Further, preliminary research suggests the acceptability, feasibility, and potential efficacy of digital interventions for NSSI. To date, however, there are few publicly available digital interventions developed specifically for young adults who engage in NSSI. The aim of this study was to solicit young adults’ impressions of early app prototypes to identify ways of improving interactive features and content needs. Building on a prior interview study which explored young adults’ self-management of NSSI and their use of technology in self-management, this study involved three waves of iterative app prototype feedback sessions with 10 young adults with past month NSSI. In general, participants responded favorably and provided feedback to augment the app to better meet their needs, including adding new features and functionality as well as increasing opportunities for personalization. We discuss two key design challenges related to the roles of tracking and temporality in digital interventions for NSSI, and then frame design considerations related to these challenges within the lived informatics model.

## 1. Introduction

Nonsuicidal self-injury (NSSI) is a global concern, affecting as many as 13% of young adults [1]. Though NSSI refers to self-inflicted harm without suicidal intent, individuals who self-injure often have thoughts of suicide and are at increased risk of suicidal behavior [2,3,4,5]. Recent longitudinal work has shown that reductions in NSSI are associated with subsequent reductions in suicidal thoughts and behaviors, providing direct evidence for the importance of targeting NSSI in suicide prevention [6]. Accessible evidence-based interventions for NSSI are a public health priority.

Though there are effective treatments for NSSI [7,8,9], most individuals who engage in NSSI do not seek formal mental health treatment. Barriers to treatment such as concerns about cost, lack of insurance coverage, and geography make treatment inaccessible or impractical for many [10,11]. Other factors such stigma, motivation, and fear of losing an effective coping strategy, contribute to high rates of non-disclosure in this population, which in turn prevent access to formal and informal support [12,13,14,15]. Thus, to appropriately address NSSI at scale, alternative models of intervention delivery which account for these barriers will likely be needed.

Digital interventions are a promising alternative to traditional in-person treatments as they are a scalable and acceptable model for increasing access to mental health services. The flexible format of digital interventions (allowing for asynchronous interactions, and various levels of anonymity and autonomy) may address barriers related to cost, stigma, and motivation. Digital interventions have also been associated with significant improvements in common mental health conditions such as depression and anxiety [16,17,18].

Early research on digital intervention for NSSI has shown the promise of app and web-based interventions as an adjunct to treatment [19,20,21]. Young people also report being interested in digital interventions for NSSI [22,23,24,25]. However, to date there are few digital interventions publicly available to young people who self-injure.

The present study builds from prior research aimed at understanding the self-management practices of young adults who engage in NSSI, as well as their current, and desired future, use of technologies to support their NSSI-related goals [26]. Here, we present findings from iterative prototype feedback sessions focused on identifying processes that young adults want an app-based technology to support, as well as the necessary and desired components of that intervention. Ultimately, the goal for this project is to develop a publicly available app to support young adults who engage in NSSI, and who are not yet engaged in formal treatment.

### Digital Interventions for NSSI

Digital mental health interventions (DMHIs) are an attractive option to reach individuals who may not otherwise engage in treatment, since they are cost-effective, scalable, and can be delivered through technologies that individuals already use daily. For example, in the U.S. 96% of young adults and 95% of adolescents report having a smartphone, with about half describing that they are online almost constantly to pass time [27,28]. Unsurprisingly, research has shown that young people who self-injure frequently seek information and support through the Internet, and they report receptivity to digital interventions [22,23,24,25].

There have been several trials of app- and web-based interventions that provide support for their acceptability and preliminary support for their efficacy [29,30]. In general, these interventions have built on, and deliver, psychotherapeutic principles from evidence-based treatments, or target novel mechanisms described in models of suicide or NSSI. Two apps—DBT Coach and BlueIce—were designed as adjuncts to in-person treatment and promote DBT and CBT skills use. In a small trial of DBT coach, app use among adults with borderline personality disorder (BPD) was associated with declines in both NSSI urges and frequency [20]. Similarly, in a 4 month open trial of BlueIce, over 70% of adolescents reported declines in NSSI behavior [19].

Web-based interventions also show promise. In the Emotion Regulation Individual Therapy for Adolescents (ERITA) program, based on a manualized treatment (Emotion Regulation Group Therapy, [31]), there was a 69% reduction in NSSI after 6 months. Like the programs mentioned above, ERITA maintains a focus on skills (emotion regulation) along with psychoeducation [21]. Experimental interventions, such as those focused on aversive conditioning and expressive writing, have produced mixed results for NSSI outcomes, and require additional follow-up [32,33]. Finally, innovative formats such as short, single-session interventions have also shown promise. For example, a 30 min online intervention for adolescents who self-harm (Project Stop Adolescent Violence Everywhere) showed preliminary support for reductions in NSSI-related targets (self-hate and desire to stop self-injury) through an randomized controlled trial (RCT) [34], and later in an implementation through the social media site, Tumblr [35]. However, this intervention was not associated with changes in NSSI behavior at follow-up.

In parallel with these recent trials, there has been an uptick in studies exploring the technology needs of this population [36,37,38]. This includes interview studies meant to elicit information about young people’s current use of technology, and desired future use. For example, in an interview study, adolescent girls [39] reported interest in a smartphone intervention focused on alternative coping strategies and psychoeducation. A study with young adults with lifetime suicidal ideation or self-harm [40], revealed user needs to store mood-lifting content, and to exchange helpful strategies with peers. Co-design workshops have also been useful to generate feedback on requirements for digital intervention [38]. A prior study upon which the current study directly builds, involved interviews with 20 young adults with lived experience of NSSI and aimed to understand how they self-manage their NSSI and their desired use of a digital tool to support them in pursuing NSSI-related goals [26]. Findings showed that young adults perceived a lack of effective coping strategies for NSSI and had sought information and support through online resources, like communities and information sites. They were generally interested in an app if it could help them to manage NSSI urges and provide them with alternative strategies to manage NSSI in line with their goals. Participants were particularly interested in drawing connections between contexts or events that may trigger a desire to NSSI, and the ability to use that information to increase self-knowledge and empower them to respond differently.

In the present study, we developed prototypes based on the needs and preferences that young adults reported in our interviews [26], to elicit feedback on app-features, functionalities, and content. Specifically, we were interested in learning about interactions that would best support young adults in achieving their goals related to NSSI, their need for specific content, and how they imagined the app would help them to draw connections between patterns, contexts, and their NSSI behavior.

## 2. Materials and Methods

This is a qualitative study using semi-structured interviews to solicit feedback from young adults with lived NSSI experience on early app prototypes.

### 2.1. Participant Recruitment

Participants were recruited via clickable advertisements on Mental Health America’s website. Mental Health America (MHA) is a national non-profit mental health advocacy group that hosts mental health screeners as well as psychoeducational resources for people experiencing mental health symptoms. After clicking on the study’s MHA advertisement, participants completed an online eligibility survey hosted on REDCap. Participants were eligible if they (1) engaged in NSSI on 2 or more days in past month, (2) were between ages of 18–24 years old, (3) owned a smartphone, and (4) were a US Citizen (due to reimbursement purposes). For safety reasons, participants were excluded if they reported: (1) severe mental health diagnoses, (2) severe suicide risk, including suicidal ideation with a plan and intent to act. All eligible participants received an email to set up a remote interview time, which also contained a link to the online consent and baseline survey with additional items on mental health and NSSI history. Nineteen initial emails were sent, from which 10 participants scheduled an interview. Just one participant that was invited for a subsequent wave after wave 1 declined due to external factors impacting their ability to participate. Interviews were conducted by KPK (PhD, MSW) over zoom, were under 1 h in duration, and were audio recorded and transcribed prior to analysis.

### 2.2. Prototypes

**Figure 1:**
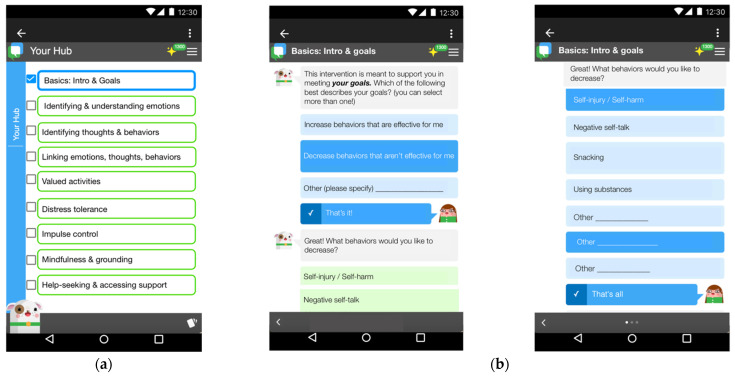
Prototypes of introductory hub and goal setting. (**a**) The hub is the main page and lists 9 modules containing psychoeducation and skills-based content. (**b**) The basics module is meant to provide users with an understanding of the app and to set and reflect on preliminary goals.

All prototypes used as part of this study were modified from the Pocket Skills’ user interface, which is an app that was originally developed by Microsoft Research that has subsequently been made available to the public (https://github.com/Microsoft/PocketSkills accessed on 29 November). Pocket Skills was designed for adults with borderline personality disorder (BDP), and preliminary field studies reported good usability metrics [25]. Using the Pocket Skills user interface as a base, wave 1 prototypes were adapted to reflect the content and functionality needs of participants from our early discovery work, and further adaptations were made at each subsequent wave. Figure 1 shows an example of the prototypes for the introductory sequence.

### 2.3. Data Collection

Three waves of iterative prototype feedback sessions were held in May through July 2022. All sessions involved a single participant and were held virtually through Zoom. The interview guide was developed following discussions among study team members who had expertise in the treatment of NSSI, human-centered design, and digital mental health intervention. Interview sessions were conducted by KPK (PhD, MSW, female) who was a postdoctoral fellow at the time of the study. KPK had prior experience interviewing young adults with NSSI and extensive training in conducting qualitative research. Participants had no prior relationship with researchers before study commencement, except for P1, P2, and P3, who participated in the research teams’ prior study which was conducted to understand young adults’ (1) self-injury self-management, (2) technology use in this self-management, and (3) imaged use of an app to assist in self-injury self-management. This study was used to generate the design of early prototypes used in the present study (for additional detail see: [26]). We had a total of 10 participants and conducted 14 total interviews which is consistent with sample size guidelines for usability [41]. Table 1 lists participant breakdown by wave.

Though data saturation occurred at around 12 interviews, the authors decided to introduce two new participants who could reflect on the final prototypes with fresh perspectives at wave 3 to ensure no new themes were identified. Following these interviews, researchers closed recruitment as saturation has been confirmed. Prototype feedback sessions lasted between 45 min and 1 h and were semi-structured to allow for probing and solicitation of feedback on newly incorporated features. During interviews participants were asked to think aloud as they viewed prompts and possible interactions within the prototype and asked questions about their needs and desired interactions with the app prototype. Prototypes were adapted at each wave to better reflect the needs participants expressed.

### 2.4. Data Analysis

All prototyping sessions were audio-recorded via Zoom and transcribed. Data were analyzed through thematic analysis as described by Braun and Clarke [1], which included six steps: data familiarization, identification of individual codes, grouping codes into preliminary themes, reviewing and refining themes to reduce overlap, defining and naming the final themes common across the whole dataset, and selecting examples from the data to accurately illustrate each theme. After several rounds of iterative open coding, the code structure was discussed among authors for face validity and conceptual clarity, and the remaining transcripts were coded. Codes were organized into a candidate set of themes which was refined to remove redundancy and retain only highly prevalent themes. All coding occurred in Dedoose software. This study is reported in line with the COREQ consolidated criteria for qualitative research [42].

### 2.5. Participant Safety

Procedures were developed to ensure participant safety throughout the study, including at baseline and during the interview. During the online baseline assessment, suicidality was assessed by self-report using the PHQ-9. In our protocol, participants scoring greater than or equal to 1 on the PHQ-9 item #9, would automatically receive item #9 from Beck Depression Inventory (2nd edition), which assesses suicidal intent. If participants scored above a 1 on the Beck Depression Inventory, an automatic email message would be sent to the first author (KPK) who would reach out to the participant within 24 h via phone to conduct further evaluation including issuing the Columbia Suicide Severity Rating scale, followed by safety planning, and, if deemed necessary, recommendations for action to ensure participant safety (including health and safety checks, calling emergency services). While there was no formal assessment of mental health symptom severity or suicide risk during the interview portion of the study, any indication of suicidal ideation or intent would initiate the same procedure described above, beginning with evaluation of risk through the Columbia Suicide Severity Rating Scale. All participants were additionally informed that their participation was voluntary and that they could pause or discontinue the interview at any time without consequences. They were also informed that they could skip questions if they felt uncomfortable. No participants scored above the risk threshold via self-report assessments at baseline or indicated suicidality within the interview, therefore there was no need to conduct follow-up phone calls or initiate the Columbia Suicide Severity Rating Scale in the interviews.

### 2.6. Participant Characteristics

Participant characteristics are listed in Table 2. Participants (*n* = 10) reported variation in past year frequency of NSSI (M = 202, SD = 184.73, range 25–497), with cutting (7/10), scratching (7/10), and hitting (7/10) as the most common forms reported. Average age of NSSI onset was 14 years old (range 11–20). Six participants reported that someone knew about their NSSI (4 reported that no one knew). Three participants made a prior suicide attempt. All but three participants reported having received mental health treatment previously, with just two reporting current therapy. On average participants reported moderate-severe depressive symptoms (PHQ9 = 14.4; range: 4–24 where 5–9 = mild, 10–14 = moderate, 15–19 = moderately severe, and 20–27 = severe).

## 3. Results

Findings are organized under seven umbrellas corresponding to areas for design focus based on participant’s expressed needs: (1) Adequate support for personal goals, (2) Flexibility in NSSI prompts and tracking, (3) Connection to crisis resources, (4) Supporting needs as users’ relationships with NSSI change, (5) Aggregating data for recommendations and pattern recognition, (6) Social content needs, and (7) Aesthetics and interactions. References to participants within text will be labeled by their participant number (e.g., P1). A summary of findings can be seen in Table 3.

### 3.1. Adequate Support for Personal Goals

One of the key findings from our interviews was the importance of goal personalization. Participants unanimously wanted to define their own goals within the app, but they appreciated scaffolding to support decision-making including the ability to choose from prepopulated behaviors or to write in specific behaviors if they were not yet represented (See Figure 1 above for goal sequence). The mix in response options was perceived to reduce feelings of overwhelm among participants who struggled to articulate their goals. Though all participants described a goal related to their NSSI behavior (e.g., reducing severity or frequency), they also identified with other mental health goals and wanted the ability to work with multiple goals at once. Specifically, participants commented on goals related to: (1) positive behaviors, (2) personal health behaviors (eating, addiction, and exercise) and (3) improving interpersonal relationships. Participants differed in the level of specificity with which they defined goals and their certainty about which goals to pursue.

As part of the goal-setting sequence, participants described behaviors that they would like to increase or decrease (See Table 4 for list of behaviors discussed). Most participants felt that their goals could easily fit within these two categories (increase or decrease), however two participants wanted to focus exclusively on positive behaviors. On this P1, described: “*I guess I personally would want to focus on more—increasing positive goals–– I guess I would just feel like more motivated if it was focusing on things I did well or ‘correctly’ instead of things I did wrong.*” Rather than having the app remind them of NSSI or other behaviors that they felt they “*shouldn’t do,*” P1 wanted the app to highlight their efforts towards improved mental health. As a way of reframing a negative behavior in a positive light, they later described: “*I can isolate a lot as a negative behavior. So, sort of turning that on its head and making it a positive behavior to increase, kind of, socializing could be helpful*” (P1).

Eating behaviors or disordered eating, addiction, and exercise, were also a common focus for goals. For example, one participant noted “*I was thinking about those negative things that you wanted to decrease. One of them was snacking, but what about the opposite? Restrictive eating is also something that would go in that same area. I feel like that is something that people who self-harm would also struggle with*” (P6). Goals around self-esteem, self-talk, and body regard were also commonly endorsed. For example, one participant described developing self-love like this:

*“Self-care things. Maintaining, I don’t know, health of my body, taking my medicine, brushing my teeth, taking a shower. So, helping me be on top of improving and maintaining my personal health, ‘cause I sometimes stopping doing those things is another form of self-harm. Not as immediate, but over time.”*

(P2)

Though many of the goals participants discussed, and the goals that were listed in the prototypes, required intrapersonal work, participants also described goals related to interpersonal relationships and help-seeking. When looking at the options, one participant described: “*I see it says frequency of negative emotions, that’s maybe how you’re thinking of yourself, and maybe you wanna change about how you view others too*” (P3). Similar comments were made by participants identifying: “*how to have the tough conversations*” (P2) and “*how to confront somebody*” or setting “*healthy boundaries*” (P1) as goals they would like to pursue and they wanted the app to contain informational content on these topics.

Notably, the level of specificity at which participants set goals varied. Some participants were more comfortable with broader categories focused on a set of practices that would lead to overall improvement in mental health. For example, a participant said: “*I guess you could put self-care or something ‘cause I don’t know what people exactly are seeking for their mental health stuff. Obviously, my situation is the self-injury part, but I mean it could fall under that category of self-care because this is the same thing almost*” (P2). Another participant suggested adding “*self-esteem*” but then clarified “*I think that’s under frequency of negative emotions*” (P4). Other participants were interested in working with more specific mental states and wanted these to be reflected in the response options. One participant described:

*“There’s this fog in my brain, and I can’t feel anything, so I don’t know if frequency of negative emotions, I don’t know if that falls under that. So, if instead it was being able to—even being able to regulate emotion might be too scientific of a way to phrase it. Something like that of where you’re describing more clearly what emotion, exactly, we’re trying to target.”*

(P2)

Some participants described that having uncertainty about goals could block people who come to the app with curiosity, but who have not yet identified a clear objective for use. For example, one participant described “*if I were unsure about what my specific goal is, but I felt like I still wanted to take part in the app it might be unclear which of them I wanted to select*” (P4). Consequently, several participants mentioned the importance of being able to change goals as their needs evolved over time.

### 3.2. Flexibility in NSSI Prompts and Tracking

**Figure 2:**
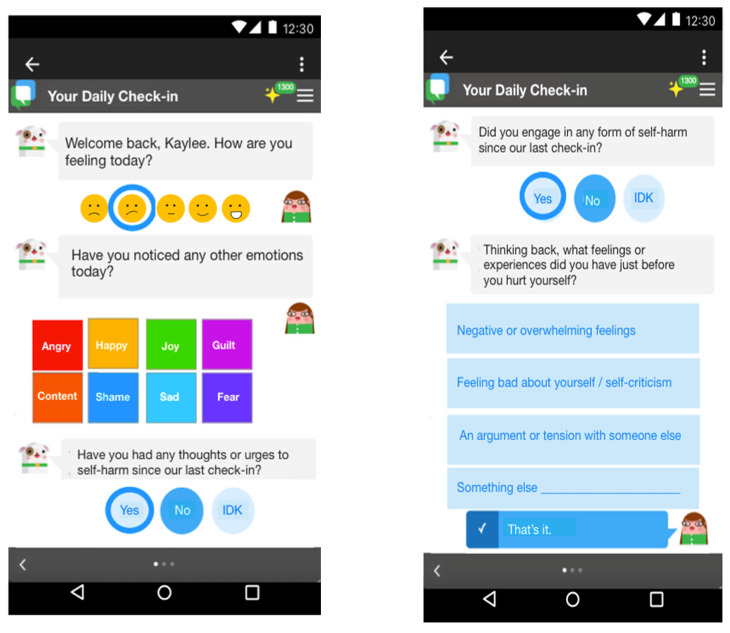
Prototypes of daily check-in. The daily check-in is meant to support user reflection on emotions and behaviors relevant to their goals.

As part of the prototype “check-in” sequence (Figure 2) participants were asked to reflect on a series of questions about their NSSI thoughts or behaviors, emotions, and skills use. The perceived value of questions specific to NSSI was highly variable across participants with some being comfortable responding to these prompts (e.g., P3 notes “*I would feel comfortable*”), and others expressing discomfort with these prompts. Discomfort was often attributed to concerns about: (1) privacy and security, (2) increased awareness of NSSI, (3) the frequency of questions. Participants who were comfortable with regular prompts felt they could: (4) bring awareness to their NSSI thoughts and behaviors and (5) provide a sense of accountability.

For several participants the discomfort stemmed from not knowing how this information would be used within the app, or who would have access to it. For example, one participant said:

*“I feel like I would be more comfortable knowing if there’s transparency, knowing that if I clicked yes, I engaged, no one was gonna be called or something, especially if you’re younger. I guess knowing that your answer is safe would be very important. So, if I felt my answer was safe, I would feel more comfortable answering honestly.”*

(P6)

Similar reflections on safety were common. However, when safety was perceived, comfortability increased: “*I would probably be pretty comfortable since I would assume that nobody else is gonna get on and see what I’ve answered*” (P2).

For others discomfort was not driven by concerns around privacy, but rather concerns about the awareness and affect that NSSI questions might evoke. Several participants noted NSSI questions may be “*triggering*” for themselves or others or may inaccurately reflect their progress. For example, one participant described “*if you’ve been working really hard and then you hear those questions and then you’re like, ‘Oh. Well, I have been still thinking about that.’ It can make you feel like it’s still a problem when it’s really not*” (P1). Others described that the frequency of reflection could be concerning: “*if I’m expected to think about it on a daily basis or a periodic basis that I think it could backfire*” (P1). They suggested making behavior or emotion tracking optional. Participants also described that longer times between NSSI questions may help alleviate discomfort. For example, as one participant described:

*“I think personally, I would be fine with a message that checks in. Because, I guess, my self-harm, [was] once in once a week or once a month where it was significant distress, that’s when it happened, and then it could be a month of nothing. So, I think from that perspective, I would be fine with it checking in a few times a week, or once a week, or something like that. Because I’ll have weeks where it’s like, “I’m good.” I see why someone who’s maybe dealing with that urge constantly, or what it feels like, they’re always trying to hold that back, I can see how for them it would be triggering then to have a reminder of it.”*

(P7)

In reflecting on comfort or discomfort with these questions, participants naturally described the ebbs and flows in their NSSI behavior over time. For example, P1 said “*I think depending on the severity, I feel like that could be kind of triggering for somebody. If they’ve been really working on not doing it and then they see those questions, it’s kind of like another reminder and kind of bring them back to that.*” As another remedy, several participants envisioned a checklist which de-centralized the focus on NSSI thus making check-in tasks feel more routine: “*Just having it as a list sort of would get less directed towards thinking about that specifically. It’s sort of more of a running your mind through the past few hours, or days, or weeks, or however often, rather than a notification or a reminder that I’m struggling with [self-injury]*” (P7).

Several participants thought they could benefit from receiving regular questions about NSSI, as a form of accountability and increased awareness. When asked about how she imagined a check in, P6 said “*at least for me, if I know that I’m gonna be asked if I did this, if I had the urge, I feel like it’s easier to not if I know someone’s gonna ask me because I wanna be able to say no.*” Others similarly felt that regular prompts were valuable because “*then you’d be able to get those responses in time, before you numb it out, or forget what led you to that point*” (P3). A couple of participants described extending the NSSI check-in to ask about suicidal thoughts—as the two commonly co-occurred in their experience. For example, a participant suggested adding the question “*are you currently having suicidal thoughts at all? Any behaviors?*” and then providing “*prompts or tips where you can talk to somebody if you feel that you need to talk to somebody in person, if it’s a really bad day*” (P3) to follow-up on any affirmative response to a NSSI question, imagining that it would be a good way to connect individuals to skill or additional resources. For participants who valued the regular check-ins on NSSI, they expected that the check-in data would help them to connect to needed resources, problem solve, or discern patterns.

### 3.3. Connection to Crisis Resources

All participants mentioned a need for the app to provide a clear pathway to crisis services. This included in-app (1) links to crisis resources and (2) easy (barrier-free) access to skills. Participants had varied experiences using crisis services like text- or call-lines but even those with no personal history with these services felt it was important to include links. They discussed several ways crisis resources could be implemented in the app itself. Some discussed “*checking if the persons in crisis*” (P1) or a “*general safety check*” (P2) when users come to the app. Similarly, others suggested a button on the landing screen to connect users to crisis sources. For example, P3 said “*At first encounter it is important to check in on the person if they are in crisis*” (P3). Another participant noted: “*There should be a place for if you are in crisis so you know who to call or have the number for the textline and a few ideas to help calm you down that you don’t have to dig for*—*a quick way to access crisis stuff*” (P8).

In general, it was important for crisis resources to remain visible on all screens when navigating through the app. P7 described an innovative way to link to crisis services that may normalize reaching out for help and enable easy access:

*“[It could be] a pop up in the corner so it’ll show a speech bubble and the bubble can go away but the button stays in case something later on in the app is triggering. That option is right there. Turn it more into a comforting presence. I’m envisioning 4 tiles, like daily check in, take me to a skill, take me to learning resource/module, and [in] the bottom left a larger version of the distress icon. But every time you open it you know its there.”*

(P7)

Given that the app would contain access to crisis services and personal data on NSSI and other behaviors that they may not want others to have access to, participants brought up specific concerns around privacy and safety with crisis features. Some participants thought it’d be important for the app to have an easy exit, like other crisis platforms (e.g., “*something where you can hide that you were on that website very quickly*” P7) and for the app to be designed in a way that concealed its purpose. This ambiguity needed to extend to notifications and other communication coming from the app (e.g., “*I’d assume the notifications would be confidential sort of, of they wouldn’t say necessarily what they are related to*” P7). Many participants noted that they did not want the app to lead to accidental disclosure or for others to judge them if they were to see mental health, NSSI, or crisis content.

In addition to having access to crisis resources, participants wanted to have easy access to skills. Potential barriers to skills, such as sequenced interactions where a participant may need to complete a daily check-in or unlock a module before accessing skills content, were an area of concern. For example, P6 described having easy access to “*few ideas to help calm you down that you don’t have to dig for, I guess. Like deep breathing, or meditation, or something, maybe have a few meditation videos. I don’t know. Just a quick way to access crisis stuff.*” Participants thought the app could be most useful to them if in-app skills were accessible with one or two touches. As such, some participants felt it should be one of the first things they encounter on the home screen.

### 3.4. Supporting Needs as Users’ Relationships with NSSI Change

When reflecting on contexts for app use most participants imagined themselves using the app around NSSI events, based on both (1) past use of apps as well as (2) imagined utility. On this, one participant described that they would gravitate to the app: “*right before I was about to self-harm, or right after*” (P2) and another describes that the app would be most useful when they have an urge or in the aftermath “*Just because you’re looking for the coping mechanism when you have the urge. And if you don’t want to do said urge, it’s good to have something to fall back on. But at the same time, when the whole event is over, it be nice to have something kinda like as a support system that’s not an actual person*” (P2). As such, their preferences regarding the frequency and utility of check-ins varied based on their current experiences of NSSI thoughts or behaviors in daily life. They recognized that their needs for contact may vary over time. For example, P2 commented on check-ins, saying:

*“So, if [self-injury] happens once a week, then I’d probably do it once a week. If that happens once a month, I’d probably do it once a month. If that’s happening every day, then I’d probably check in every day. But I’m assuming my thought back to the app would probably only happen, like I said, right when I’m about to or right after I have. So, if it’s right after I have, it’s probably to get myself back on track, wanting change. If it’s right before I do, it’s probably last minute hope to change my mind.”*

(P2)

Notably, participants’ experiences of NSSI frequency varied—with some having long periods between NSSI events. Though participants could most easily imagine themselves using the app when NSSI was salient, they wanted the app to be valuable to them independent of NSSI events. As such, they wanted the content of check-ins and skills practice to be generalizable to periods when they were focused on general well-being. When participants envisioned the app’s purpose and potential function as more than supporting them with NSSI goals—e.g., supporting skills practice—they envisioned more frequent contact. In these cases, check-ins were perceived to be most useful feature to sustain participants engagement with the app, and a reminder of their goals.

Many participants could see the value in skills practice daily, but they wanted to have autonomy in when they would engage in skills practice. For example, one participant said: “*I think having some of those practice options daily would be a good place to start. Even if it’s a five-minute thing, just a short, daily thing that it recommends for you*” (P3W1). However, daily skills practice wasn’t valued by all participants, and some felt that reminders to engage in skills would irritate them over time. Nearly all participants suggested making these reminders optional and customizable. For example, one participant described: “*some people hate getting daily reminders, so I do think it should be optional rather than just an automatic thing. But for me, that would help me at least remember that this app is there every day, so I don’t forget about it until I’m in crisis mode*” (P2).

As an alternative to reminders, another way participants imagined engaging in skills practice was to bookend check-ins and modules with opportunities to practice skills with the app’s support. For example, one participant described that if a check-in “*leads me automatically into another thing, I’ll do that other thing. Yeah, so maybe saying, “Do you want to learn—Do you want to get started on this new module or try this new activity”*” (P1). If such a transition wasn’t built into the app, participants thought they would forget to use skills in their daily life.

### 3.5. Aggregating Data for Pattern Recognition and Reflection

In initial interviews, young adults reported wanting to identify and reflect on patterns related to their NSSI and to receive recommendations that would help them interrupt harmful patterns and improve their mental health. Participants imagined that this could be facilitated in two ways: (1) an automatic detection and recommendation system and (2) data aggregation that enabled reflection.

Many participants assumed the app would have internal logic to make smart recommendations on skills practice based on their prior data. For example, one participant imagined the app using emotion data from the check-in to suggest specific skills for in-the-moment use: “*coping skill suggestions based on emotions—emotional identification and then coping skill. Like, you’re feeling this way, here’s what you can do versus you’re feeling a different kind of way, you can do this instead. I think that could be helpful*” (P1). Similarly, a participant envisioned a specific section in the app that could recommend practices or activities based on user-inputs, drawing a comparison between this imagined functionality and other systems they use: “*Like music does it if you listen to certain songs. They recommend it… you could put like recommended activities*” (P2). A smart system was perceived to be useful largely because it would “*track your habits … or your patterns and then remind you of them*” (P1) which empowered the user through increased self-knowledge.

Other participants shared the desire for data to be aggregated so that they could reflect on it and discern patterns themselves. On this, one participant discussed ways that the check-in and NSSI data could be stored so that it could be easily searchable later:

*“If they’re able to, over the course of the app, identify why they self-injure, I think that’d be really helpful—I wonder whether if they (app users) notice they’re self-injuring, would they be able to go to the app, and be able to be like, “I did this, what am I feeling right now, what did I feel after I self-injured?” Because normally, that type of reflection only happens consciously way after. But being able to do that closer to the moment might help them identify why, and then identify how to stop better.”*

(P3)

Participants were interested in reflecting on more than the check-in content—including free-text that they input into the app through notes or prompts within modules. Many participants described writing to understand their feelings, for example, “*I often write things down in a notes app when I’m thinking about things. And then I kind of, pretty often, just go back and flip through. Like, what did I write at this time and things like that*” (P4). Participants were inclined to store typed content and aggregate it for later use. On this, one participant imaged that the app would compile notes “*so then that way it’s easier for me to cross reference something specific that I wrote next to each other rather than having to go to different sections to look for it*” (P4). A similar functionality was imagined for skill use. For example, one participant said: “*I think having that ability to sort of keep track of the skills used, and when you were doing behavior that you’re trying to stop, I think that would be pretty important*” (P7). Another participant thought the daily check-ins could be an opportunity to understand what skills were most effective for them. She described an extension of this:

*“Maybe also have, ‘Did you engage in any form of self-harm since our check in?’ [If] ‘No’ ‘Were you tempted to engage in any form of self-harm since our last check in?’… Then, maybe if they say they were tempted say, ‘Okay. You were tempted, what stopped you from actually going through it? Did you use these skills?’”*

(P2)

Other participants described saving emotion-related content and looking at associations between emotions, NSSI, and skills. In sum, the ways in which participants wanted to receive information were nuanced and should be explored in future work.

### 3.6. Social Content Needs

Participants wanted more information on: (1) maintaining healthy relationships and boundaries, (2) having conversations about NSSI/mental health, and (3) testimonials about other people’s experiences in recovery. Notably, these three topics are social by nature, whereas topic areas presented to participants in as part of the prototypes were mostly intrapersonal (e.g., emotion regulation, mindfulness).

Several participants described that NSSI thoughts and behaviors stemmed from difficult social interactions. As such, they wanted to learn how to maintain healthy relationships, including defining and maintaining appropriate boundaries. For example, one participant described: “*I know that for me, that’s where most of my anxiety comes from—other people. So, providing support for that, and showing resources, also how to be more confident when interacting with others, and how to handle some of those tricky situations would be really helpful*” (P1).

Relatedly, participants expressed difficulty having conversations with others about their NSSI, and other mental health struggles due to fear, stigma, or shame. Several participants mentioned that it would be helpful to have information or even a template for how to start these conversations with different people in their life. For example, P2 said: “*How to have the tough conversations—‘cause I think a lot of people when they have a bad habit it’s kind of a thing of shame for them.*” This participant felt it was important to have “*different options on there would be how to talk to your parents about this, how to talk to a significant other about this, how to talk to your friends about this, how to tell your teacher or boss at work that you need help with this…*”

Finally, some participants felt it would be important to hear from others who had used the skills the app taught, to establish the credibility of the skills and relatability of peers’ experiences. One participant described “*I think it’s helpful—to hear some common things that seemed to work for other people; it might not work for you, but maybe it will*” (P1). Testimonials needed to be honest and transparent—reflecting both things that worked and things that did not work for peers. Another participant describing the value of hearing others’ stories, imagined that this information could be shared via video and make her feel less alone in working towards her goals “*having people speak to their experiences in video format, I think would actually help. Because you’d have suddenly a personal interaction, even it’s not directly at you, you’d be reminded that other people are going through this, and other people have overcome it*” (P3). In sum, interpersonal strategies and help-seeking resources were desired.

### 3.7. Aesthetics and Interactions

While we used the prototypes mostly for elicitation and ideation, the feedback participants provided regarding the user interface was useful and overwhelmingly positive. In general, participants commented on (1) the language the chatbot used, (2) the built-in incentive structure and (3) desired customization. Several participants commented on the interactive feel the chatbot afforded. For example, one participant said: “*I like that it was interactive, and it would ask you questions, and then when you answer it, it gives you good feedback. I love that*” (P3). Participants also referred to the human-like style of interaction: “*it doesn’t seem super professional, so it seems more personable*” (P6) and appreciated that there were “*little humorous remarks in there*” (P4). Participants liked language that was supportive and direct but noted that a few interactions “*didn’t sit right*” because they felt dis-empowering or “*insensitive*” (P3). In these cases participants felt the chatbot’s language didn’t clearly acknowledge their agency.

Participants also provided feedback on the incentive structure in the app—which involved gamification through being rewarded with stars when they completed a module, skill, or check-in. Stars could then be used to unlock new content like skills practices, or videos. On this, one participant said: “*I think the –daily reward and the stars…I think that makes sense. I play a lot of games where you kind of do it every day, and it’s nice as some type of structure. And so, while this isn’t a game obviously, it’s still kind of, I think an easy to add it to a routine*” (P4). Another participant described: “*I’m very extrinsically motivated sometimes. So, I think that would be helpful for me to know, oh I have to do my daily check-in, or I won’t get this reward, whatever it is*” (P1). Though an incentive structure was perceived to be necessary but did not need to be complex.

Participants also wanted to be able to customize the avatars when they first set up the app. Some imaged that avatar customization could be part of the incentive structure by allowing users to change features of the avatar or buy accessories for their avatar as a reward for using the app. One participant described “*I was also thinking with the stars and being able to change the avatar kind of thing. What if you could also change the dog? That could be something you could buy as well*” (P6). Similarly, another participant described the value of turning in points for avatar accessories like this: “*I’m actually doing something good, and I can physically see it.” Like, “Spot started with a small bed, now he’s got a cute bed, and a cute beach set up. I’ve been saving up and doing my daily check ins so I can get him this cute dish—it’ll keep you coming back*” (P3).

This participant also noted that it can be hard for people to do things that are good for themselves, but that the app could trick “*you into trying what’s good for you…, even if you’re not putting yourself first, you might put a little cartoon dog first, but it’s helping you in the end*” (P3).

## 4. Discussion

In this study young adults with recent experiences of NSSI provided feedback on prototypes meant to solicit information about their needs and preferences for an app to support them in their NSSI self-management. In general, participants responded positively to the app prototypes and provided feedback on ways to augment the functionalities of the app and diversify content, so it better met their needs. We devote the discussion to findings that raise challenges to the future design of an app to support this population with the intent of underscoring key considerations for stakeholders working in this research space. Specifically, we explore the roles of tracking (Section 4.1) and time (Section 4.2) in digital interventions for NSSI and discuss design considerations and possible solutions.

### 4.1. If and How to Incorporate NSSI “Tracking” in an App

The first challenge introduced by our participants was related to the value and necessity of tracking to support NSSI self-management. Young people with lived experience of NSSI often describe wanting to understand contexts and patterns related to their NSSI behavior so they can better anticipate and prevent future episodes. In our study, like others [38,39], they imagined that an app could support them in this endeavor, yet their comfort with tracking NSSI was highly variable. Some participants were comfortable with receiving app prompts asking them questions about NSSI but wanted to have control over how often they received these prompts. Others described contextual factors that would influence their comfort, including their current experience of NSSI (e.g., the frequency and severity of thoughts or behaviors) and the way in which prompts were presented (e.g., checklist vs. direct question). Though some degree of tracking will be necessary to support the pattern recognition that young adults’ desired, the potential for unintended harms, as expressed by participants, foregrounds questions around ethics and safety. Below, we briefly review literature from the clinical, research, and design domains on tracking to explore solutions and identify areas in need of further consideration.

From a clinical perspective, self-monitoring of NSSI is a core component of treatment. Most treatments incorporate a functional assessment of NSSI and ongoing tracking of NSSI thoughts and behaviors as well as antecedents and consequences associated with the behavior. For example, chain analysis in dialectical behavior therapy (DBT) engage patients in creating a detailed sequence of events to identify vulnerability factors (emotions, external environment, thoughts) associated with their NSSI [43]. Other cognitive-behavioral treatments use similar techniques such as daily diaries and mood or self-harm charts to document behaviors, thoughts, and contexts over the course of treatment with the ultimate goal of reflecting on patterns surfaced to increase awareness and develop insight [44,45,46,47]. To facilitate these techniques patients are necessarily asked to think about, and record, their NSSI behavior.

The effects of repeated prompting have similarly been explored from a research perspective. Technological advances have provided opportunities to study NSSI in daily life through intensive longitudinal studies. Many of these studies use ecological momentary assessment methodologies, which involve asking repeated questions about NSSI over time, in order to understand risk factors and opportunities for in-the-moment intervention (e.g., [48,49,50,51]). Several studies in this research space have shown that frequently assessing suicidal thoughts is not associated with a change in the severity of suicidal thoughts—though these studies did not measure NSSI specifically [52]. Together clinical and research evidence shows that repeated assessment of NSSI in service of tracking thoughts or behaviors is common practice and does not raise significant concern around harm.

In contrast, in human–computer interaction (HCI) studies of self-tracking technologies it is not uncommon for close monitoring to exacerbate symptoms [53], and ultimately result in the abandonment of tools that could otherwise help [54]. This is a concern our participants voiced about unintended consequences. For example, in a study that involved tracking stress, data increased user awareness of stress, and exacerbated stress for some [55]. Similarly, in an interview study [56] with people with multiple chronic conditions, self-tracking was perceived to be a burden and it emphasized symptoms that made them feel worse about their condition. Though the benefits of tracking have also been noted in HCI for chronic condition management and behavior change [57,58].

Another important factor to consider is the differences between tracking activities within clinical or research environments (including design research environments), and the environments where an app may ultimately be deployed. These difference merits careful consideration when designing a resource for a potentially high-risk and vulnerable population. For example, within traditional treatment settings, when the clinician explains the purpose of these exercises they can gauge patient comfort, offer appropriate support, and modulate any reactions as part of the therapeutic alliance. Clinicians also review tracked data with the patient and guide them towards personalized solutions to interrupt harmful cycles. Though digital tools have functionalities to provide psychoeducation, support, and offer personalized solutions through recommender systems [59], their capacity to gauge user comfort and modulate user reactions is weak. Low-intensity coaching, wherein a human coach provides a user with minimal support around goals and motivation, offers one solution [60,61,62], yet there may be cases where a human-in-the-loop design is not desirable or feasible.

There are also differences between research environments and direct-to-consumer environments where many apps are deployed. This is especially true for projects that involve high risk populations, which include extensive safety monitoring and protocols that detail thresholds for escalation ranging from automatic messaging to study team outreach, to deploying emergency services [63]. These protections can be mirrored in human-assisted digital interventions since the key difference between implementation of the tool within a research study, and the natural environment, is extra support provided by research staff. However, this level of oversight is not possible for standalone interventions where there is not a clinical monitor. In these cases, safety features—such as buttons that provide direct access to crisis lines, as well as informational briefs on where and when to seek help—are essential.

In sum, evidence from these different domains suggests the potential value of incorporating tracking functionality within an app for NSSI, but it also underscores the need to design with unintended harms in mind and allow customization. To date there has been little guidance on what support should be in place to accommodate for differences in digital interventions meant to be deployed “in the wild,” and those that are being evaluated and deployed within clinical or research settings. As efforts to develop publicly available digital tools to support young people who self-injure grow, such guidance will be critical.

To that end, our participants provided useful insights on solutions to address variability in comfort. For example, several participants described that a checklist of behaviors or outcomes would feel less confrontational than a direct question about NSSI, and therefore be less likely to cause harm. From a design perspective, this would enable collection of data about NSSI which could be valuable to rendering patterns for users. Other participants suggested that the app have options to select the frequency of questions, and to turn questions on, off, or to pause questions. This would provide users with autonomy and empower them to use the technology in the way it serves them but would potentially impact the ability of the app to render useful feedback on NSSI. One way to accommodate this option may be to track antecedents (triggers) rather than the behavior itself.

### 4.2. Considering Temporal Features in Apps for NSSI

The second set of challenges surfaced by our participants was related to time. Participants described their experiences of NSSI with temporal caveats and demarcations, which subsequently impacted their needs and the support they desired from an app. An app would need to be sophisticated enough to render tracked behavior on a timeline that was meaningful for the participant, while also being responsive to participants’ needs as they changed over time. To outline how temporal factors may influence the design of an app for this population, we adopt a framework that describes three temporal features [64]—temporal trajectories, temporal rhythms, and temporal horizons—and highlight how they emerge from, and influence, young people’s experiences of NSSI self-management.

#### 4.2.1. Temporal Trajectories

People’s experiences of NSSI self-management are often conceptualized along a recovery trajectory with different stages [65,66]. For example, an application of the transtheoretical model of behavior change (TTM) to NSSI characterizes six stages that are differentiated by an person’s readiness to make changes in support of their desired outcome [65]. At each stage, shifts in cognitive, behavioral, and emotional processes (or mechanisms) contribute to progress [67]. Typically, as people move from early to later stages in the process, they shift from thinking and learning about their behavior (cognitive processes) to taking actions to change their behavior (behavioral processes) [65,68,69]. Though stage-based models provide a framework to show the many common factors that contribute to this process, they are not a perfect conceptualization of recovery. Indeed, as our participants noted, personal trajectories of NSSI recovery are *not linear* [70,71,72], and the change desired is not homogenous or static [65]. People can report many goals related to their NSSI (e.g., to change frequency, reduce severity, to stop), they can prioritize these goals differently depending on context, and these goals also change over time [26].

Like recovery trajectories, which refer to a person’s progress towards their desired goal, temporal trajectories can be a useful concept because it can focus researchers and designers to consider how an app can support users’ current needs while simultaneously collecting data relevant to supporting future needs, and to tailor content accordingly. The types of therapeutic content and goals that most resonate with a user are likely to vary based on where they are in their trajectory. Similarly, technology features may also need be conditioned by temporal trajectories—users in later stages may be more receptive to direct prompts, nudges, and behavioral activation from an app, whereas people in earlier stages may be most interested in learning and engaging in cognitive processes (e.g., psychoeducation and monitoring to increased awareness). We note that while our participants spoke to the fact that there are different needs at different points in recovery, and with different experiences of NSSI severity, they were not likely representative of all stages so our ability to speak to specific needs across the spectrum are limited but merit future work.

From a design perspective, another consideration that must come early in app development is whether the tool is meant support users at all stages of their recovery trajectory, or whether it will focus on one or several stages—which shortens the temporal trajectory for design. For example, if a tool is meant to be adaptive to needs throughout recovery, then it will likely need to incorporate more sophisticated functionalities, like automated tracking, to detect and account for changes overtime. Features may also be needed to allow users the flexibility to go back to work on goals that were previously met, to change targets for goals, and to adjust needed levels of support and/or contact. However, there may be cases when a shorter, more targeted intervention is desired.

#### 4.2.2. Temporal Rhythms

Participants consistently reported the desire to understand, and better intervene on, patterns related to their NSSI. These patterns—or rhythms—provide structure to individual experiences of NSSI even when the specific sequencing of these rhythms were not always well understood by participants. Here, we focus on two temporal rhythms: episodes and events.

NSSI can be episodic—people can go weeks or months without any NSSI behavior, and then self-injure frequently during other periods. Participants ideas about how an app could support them differed significantly based on whether they were currently experiencing an episode of NSSI (used here to refer to a period of frequent NSSI). It would thus be useful for a technology to collect data about these episodes to adequately support user needs. Given participant reservations with direct questions about NSSI, it may be worthwhile to explore more acceptable ways of gathering necessary information, such as through passive sensing, or inquiring about orthogonal factors related to goals [73]. It may also be that a person’s comfort with direct questions about NSSI differs based on whether they are experiencing an episode, and where they are at in their trajectory. As such, it may be useful for a technology system to be able to respond dynamically to all three temporal factors.

The second rhythm relevant to participants’ experiences of NSSI was characterized by the sequence of factors leading up to, and following, NSSI events. An event is meant to convey one instance of NSSI behavior. Event rhythms are the subject of the cognitive-behavioral techniques described in Section 1 (e.g., chain analysis, functional assessment), and include the sequencing of NSSI thoughts, behaviors, urges as well as antecedents and consequences. For an app to support users in gaining a better understanding of their patterns, it will need to capture data relevant to this sequence. From a design perspective, it is important to think about the role this event data would serve choose a data collection strategy that is acceptable and minimally invasive. Because participants wanted to reflect on the data themselves, and for the data to inform personalized recommendations for how to manage distress in the moment, both visualizations (such as graphs) and textual feedback (insights or recommendations) will likely add value. Additionally, the type of support and messaging perceived to be beneficial to a user before or after a NSSI event may differ. For example, our participants described that distraction techniques within the app (e.g., distress tolerance [43]) would be useful when they have an NSSI urge to, whereas reflection techniques and supportive messages, may be more valuable afterwards.

#### 4.2.3. Temporal Horizons

Finally, temporal horizons refer to how individuals self-manage NSSI on a day-to-day basis and are conditioned on both temporal rhythms and trajectories. A simple example of a design implication related to temporal horizons comes from our understanding of chronic condition management. Specifically, when energy is depleted users may prefer, and be most capable of, focusing on short-term goal in service of meeting a longer-term goal [73,74]. Participants described horizons when they discussed their desire for immediate in-the-moment activities to distract on days when they were having active urges, in contrast to content on new strategies or reflective exercises on days when their distress was lower, and their horizon was longer. An app should be able to adequately support users when their temporal horizon shifts to maintain user motivation and meet their needs. As an example, this could include offering a series of decision-making prompts to understand whether users have a short or long horizon while setting daily intentions and adjusting check-ins throughout the day to meet them where they are. Similarly, a focus on temporal horizons can also help researchers and designers build the functionality needed for users to be able to set, and work with, shorter-term (e.g., daily, weekly) and longer-term goals (e.g., monthly, yearly).

#### 4.2.4. Temporal Features CAN Guide Design

The concept of temporality, including trajectories, rhythms, and horizons, focuses our attention on the how people manage NSSI over time and provides appropriate context to understand the activities that they undertake when self-managing. Consequently, these three features can help us to identify, and design for, unique challenges or barriers that may impact user experience under different temporal conditions. In the final section, we provide an example of how temporal features can be considered when making design decisions. We frame the example within the three stages of user experience outlined by the Lived Informatics Model: (1) deciding and selecting (user onboarding: choosing why and how to track); (2) tracking and acting (sustained use: collecting, integrating, and reflecting on, data), and (3) lapsing and resuming (breaks: pausing, and returning to, tracking) [73].

**Deciding and selecting stage.** Users come to technology decision points—e.g., whether, when, and how to use a technology—at particular stages in their recovery trajectory, with unique temporal rhythms and horizons. Recognizing this, and meeting a user where they are at when they first engage with a technology, has the potential to improve initial uptake, increase engagement, and ultimately result in more beneficial use. As an example, when users are first introduced to an app, they could be presented with a series of prompts to assess where they are in their temporal trajectory—e.g., their desired outcomes, readiness to change, and preferences for cognitive or behavioral strategies. Simple decisional logic could be used to determine a provisional sequencing of modules to prioritize certain psychotherapeutic content, and tailor in-app interactions to build on their existing strengths. This sequencing could be refined or updated over time as more data is collected through daily check-ins and general app use. In this case, automated learning—a method is used frequently for recommendation systems and to deliver personalized insights to users within digital mental health interventions [59,75]—may be necessary to tailor content based on temporal rhythms and horizons.

**Tracking and acting stage.** Once a user has chosen to get started with a technology and begins use, they transition to the tracking and acting stage. In this period of more sustained use, participants described interest in reflecting on, and learning from, data as well as receiving recommendations for how to manage distress in the moment. These two needs corresponded directly to temporal rhythms; therefore, the tool must be able to collect and display data related to episodes and events. Common ways of tracking within system design such as displaying frequency of NSSI along a timeline or days without injury as a count variable were not perceived to be valuable and may be harmful. Instead, they imagined a program to collect and aggregate a wide range of data so they could look at longer term patterns (e.g., trajectory, episodes), as well as more granular detail about thoughts, emotions, skills, and behaviors relevant to their goals at a daily level (e.g., events). To accommodate this, data from daily check-ins may be used to discern patterns, as it has been used in prior EMA work connecting signals like affect and interpersonal conflict with risk of NSSI behavior [49,50,76]. Manual (self-report) entry may be acceptable for users who are highly motivated and comfortable with more frequent questions, however it also places a heavy burden on the user, so passive sensing is a worthwhile alternative to explore.

Participants were also interested in learning and doing—which meant engaging with psychoeducational content and practicing new strategies. They also wanted feedback on the types of strategies that worked best for them and to have an easy way to search their own data. Detailed feedback on historical data was not perceived to be useful when they were actively in a NSSI episode, however. A consideration of temporal horizons, and users’ capacity to focus on longer versus shorter-term goals, can help guide the delivery of content at this stage.

**Lapsing and resuming.** Finally, the lapsing and resuming stage may be most important in designing a tool that is appropriate for the behavioral trajectories for NSSI, and often receives the least attention in the design of digital interventions. Users may forget to use the app or stop using the app for a variety of reasons including difficulty managing upkeep, or intentionally skipping entries or suspending use when NSSI has improved or, conversely, when NSSI is more frequent. Designing for these natural rhythms may help mitigate unwanted lapses and increase the likelihood of users coming back to the app at a later timepoint. Two approaches may be helpful when designing for this stage. First, participants that when NSSI behavior was infrequent, an app could be of service to them if it included content and resources for overall health and wellness. Moreover, regular engagement with this content would make the app more salient in times when they could most benefit from the app’s NSSI resources. In addition to including more diverse content, designers can also think about how to support individuals as NSSI ebbs and flows and their mental health, and subsequently app, needs change. It may be possible to detect shifts in user activity and initiate a chatbot conversation with the user to gently bring this change to the user’s awareness, and to see if they’d like to schedule a break, or to adjust parameters of use, providing user autonomy and empowering them to determine their timeline.

In sum, we have provided examples of how temporal features can inform design decision-making at each stage of app use via the lived informatics model. The overarching goal for design will be to provide individuals with enough structure at each stage to progress and benefit from the resources offered, but also to provide support and flexibility for users to choose the interactions they want and feel comfortable with. Temporal features provide important context that can inform such structure.

## 5. Limitations

This study has many strengths including directly soliciting detailed feedback from a diverse group of young adults with lived experience of NSSI. However, there are also some limitations. First, we have a relatively small sample of participants who were recruited based on narrow criteria. This does not represent a broader sample of young adults who NSSI, including those with past month suicide ideation, or those with serious mental illness. Second, just one participant was engaged in treatment, and had the ability to reflect on what would have been helpful when not treatment engaged. Future work may wish to engage more participants who have a history of treatment, and who are at different stages of recovery, as they can reflect on what specific tools from therapy were beneficial. Third, while we discuss the importance of considering recovery trajectories in design, we must also note our sample is not likely representative of those in early (precontemplation) or later stages of change (maintenance) since they self-selected to be involved in this research and discussed goals related to behavior change. Fourth, the demographic data collected at baseline is limited and future work would benefit from including categories to represent a broader range of ethnic groups. Finally, we note that males are under-represented in our study.

## 6. Conclusions

Digital interventions for young adults who self-injure have potential to increase access to evidence-based resources that are attuned to their unique needs. In this study, we conducted iterative prototype feedback sessions focused on identifying processes that young adults want an app-based technology to support, as well as the necessary and desired components of that intervention. Findings have implications for the design of digital tools and will be used to develop a publicly available app to support young adults who engage in NSSI, and who are not yet engaged in formal treatment. Needs differed both across and within participants suggesting that other contextual factors may influence a users’ needs and should be considered within the design of the app itself. We found that participant variation was not just categorical (between persons) but also dynamic and shifting with time (within person) and highlight temporal features within design.

## Figures and Tables

**Table 1:** Participant breakdown by wave.

Wave	Participant ID
1	P1, P2, P3
2	P1, P3, P4, P5, P6,
3	P1, P3, P7, P8, P9, P10

**Table 2:** Participant characteristics.

	M (SD)
Age	21 (1.59)
	N (%)
Gender identity	
*Female*	8 (80)
*Non-binary or third gender*	2 (20)
Race	
*Black of African American*	3 (30)
*Asian*	1 (10)
*White*	6 (60)
Ethnicity	
*Hispanic*	2 (20)
Diagnosis by mental health professional	
*Depression*	8 (80)
*Anxiety*	6 (60)
*Obsessive compulsive disorder*	2 (20)
*Eating disorder*	1 (10)

**Table 3:** Themes and summary of key takeaways.

Themes	Summary
Adequately support for personal goals	Adequate support for goals within the app meant balancing flexibility in terms of being able to work with goals that resonated with their experience, and met their needs on one hand, with structure, and support for goal setting on the other. Participants did not want a blank slate. Instead, they wanted to see response options that they identified with to curb anxiety and give them ideas for goal setting.
Flexibility in NSSI prompts and tracking	Participants expressed mixed opinions on their comfort and the perceived value of answering NSSI questions regularly within the app. Reasons for discomfort included safety and privacy concerns, increased awareness, potential affective responses. To mitigate this discomfort participants suggested allowing users to customize how regularly they received questions or having a checklist of behaviors they are working towards, to make it a less direct inquiry. Those perceiving benefit felt that it would promote accountability and would be useful to reflect on in combination with other data.
Connection to crisis resources	Crisis resources were perceived to be an important part of the app. These resources included easily accessible links to existing hotlines and informational sites as well as on-demand skills to practice in moments when they were overwhelmed or experiencing an urge to injure.
Supporting needs as users’ relationships with NSSI change	Participants felt app use was most likely to be anchored to NSSI events. However, broadening the app’s purpose to include other goals related to mental health, increased the likelihood of routine use. Check-ins were perceived to be valuable, but the frequency of reminders/notifications needed to be customizable.
Aggregating data for recommendation and pattern recognition	Participants wanted to identify and reflect on patterns related to their NSSI so they could interrupt harmful patterns. This was imagined to be facilitated via an automatic tracking and recommendation system to help them practice in-the-moment skills and a data aggregation and display system that would enable reflection and empower them to identify patterns themselves.
Social content needs	In addition to the intrapersonal categories represented in our prototypes, participants described emphasized the need for (1) information about maintaining healthy relationships and boundaries, (2) having conversations about NSSI/mental health, and (3) testimonials about other people’s experiences in recovery.
Aesthetic and interaction preferences	The chatbot interface was perceived to be easy to use and engaging, and participants liked avatars. The simple incentive structure (accruing stars to unlock content) was valued but participants imagined expanding upon this to include more customization including buying accessories for their avatar or making cosmetic changes to the app interface.

**Table 4:** Domains for participant goals.

Behaviors to Decrease	Behaviors to Increase
NSSI	Exercise
Addiction	Sleep
Negative or distressing emotions	Coping skills
Body image	Work productivity
Eating behaviors	Socialization
Negative feelings about body	Self-care
Self-criticism	Self-talk
Impulsivity	Time management

## Data Availability

The datasets presented in this article are not readily available because this study involves qualitative data from Interviews that is difficult to anonymize. Requests to access the datasets should be directed at: kaylee.kruzan@northwestern.edu.
